# Transient potassium channels augment degeneracy in hippocampal active dendritic spectral tuning

**DOI:** 10.1038/srep24678

**Published:** 2016-04-20

**Authors:** Rahul Kumar Rathour, Ruchi Malik, Rishikesh Narayanan

**Affiliations:** 1 Cellular Neurophysiology Laboratory, Molecular Biophysics Unit, Indian Institute of Science, Bangalore, India; 2 Center for Learning and Memory, The University of Texas at Austin, Austin, TX, USA

## Abstract

Hippocampal pyramidal neurons express an intraneuronal map of spectral tuning mediated by hyperpolarization-activated cyclic-nucleotide-gated nonspecific-cation channels. Modeling studies have predicted a critical regulatory role for *A*-type potassium (KA) channels towards augmenting functional robustness of this map. To test this, we performed patch-clamp recordings from soma and dendrites of rat hippocampal pyramidal neurons, and measured spectral tuning before and after blocking KA channels using two structurally distinct pharmacological agents. Consistent with computational predictions, we found that blocking KA channels resulted in a significant reduction in resonance frequency and significant increases in input resistance, impedance amplitude and action-potential firing frequency across the somato-apical trunk. Furthermore, across all measured locations, blocking KA channels enhanced temporal summation of postsynaptic potentials and critically altered the impedance phase profile, resulting in a significant reduction in total inductive phase. Finally, pair-wise correlations between intraneuronal percentage changes (after blocking KA channels) in different measurements were mostly weak, suggesting differential regulation of different physiological properties by KA channels. Our results unveil a pivotal role for fast transient channels in regulating theta-frequency spectral tuning and intrinsic phase response, and suggest that degeneracy with reference to several coexisting functional maps is mediated by cross-channel interactions across the active dendritic arbor.

Intricate regulation of neuronal physiological properties is a prerequisite for robust brain functioning as alteration of these properties could result in pathological conditions^1^. In scenarios where a given set of neuronal properties is exclusively mediated by a single ion channel subtype, such robust regulation of neuronal properties presents a paradox because ion channels exhibit huge variability in terms of densities, voltage dependence and kinetics owing to differences in trafficking targeted at the plasma membrane, in channel-binding interactions with accessory subunits and dynamics of intracellular biochemical milieu^2^,^3^,^4^. Multimodal regulation of neuronal properties, whereby different ion channels modulate neuronal properties that they don’t mediate, has been suggested as a resolution to this apparent paradox^2^,^5^,^6^,^7^. For instance, although blockade of sodium channels results in complete cessation of neuronal firing, channels including the *A*-type K^+^ channels have been implicated in the regulation of neuronal firing rate^8^ when they coexpress with sodium channels. Here, although sodium channels are essential for *mediating* neuronal firing, the presence of *A*-type K^+^ channels *modulates* firing rate, presenting a scenario where specific neuronal firing rates are achieved through several combinations of structurally distinct ion channels^8^. Such degeneracy, the ability of disparate channels to yield analogous function, is prevalent across neural systems and has been postulated as a substrate for robust expression of physiological phenomena^2^,^5^,^6^,^7^,^9^,^10^,^11^.

In this study, we dissected degenerate mechanisms contributing towards the regulation of functional maps associated with theta-frequency (4–10 Hz) spectral tuning properties in the dendrites of rat hippocampal pyramidal neurons. The functional map of subthreshold theta-band spectral tuning in pyramidal neurons is mediated by the hyperpolarization-activated cyclic-nucleotide-gated nonspecific-cation (HCN) channels, with spectral selectivity at hyperpolarized potentials completely abolished at all locations by blocking HCN channels^12^,^13^,^14^,^15^,^16^. Despite such elimination of spectral tuning with HCN-channel blockade, modeling frameworks have demonstrated that specific ion channels expressed in hippocampal pyramidal neurons could contribute to degeneracy in the somatodendritic expression of theta-frequency spectral tuning^2^,^7^. Specifically, these studies showed that leak, *T*-type calcium and *A*-type K^+^ (KA) channels could contribute to such degeneracy by regulating the specific frequency of tuning, whereas other channels such as the fast sodium and the delayed rectifier potassium did not significantly alter these tuning properties. Whereas the role of leak and *T*-type calcium channels in regulating resonance were consistent with previous literature^17^,^18^,^19^, the prediction that KA channels could alter *low-frequency* spectral tuning is surprising. This is surprising because low frequency spectral tuning has primarily been attributed to slow resonating conductances and not to fast transient conductances like that of the KA channel. Further, the inverse relationship between channel time constants and resonance frequency^7^,^17^,^18^ also argues against a role for the fast transient KA in sustaining theta-frequency resonance^7^. These predictions by computational studies on a role for hippocampal KA channels in regulating theta-frequency spectral selectivity, thereby conferring degeneracy in spectral tuning across the somatodendritic arbor, have not been assessed.

Here, employing somatic and dendritic recordings from rat hippocampal pyramidal neurons, we positively test the predictions engendered from computational frameworks and unveil novel roles for KA channels in regulating theta-frequency spectral tuning, intrinsic phase response and excitability measurements across the somatoapical trunk. Specifically, we employed two structurally distinct pharmacological agents to block KA channels in separate sets of experiments (to explicitly rule out established non-specificities of each blocker), and demonstrate a significant impact of these channels on sub- and supra-threshold excitability and on several impedance-related properties across the neuronal somato-apical trunk. Assessing intraneuronal changes, we also show that correlations between percentage changes (after KA channel blockade) in different measurements from the same neuron were weakly correlated, suggesting that different physiological properties are differentially regulated by KA channels. Our results clearly demonstrate that degeneracy with reference to different neurophysiological properties is mediated by active spatial and kinetic interactions between different ion channels that express across the somatodendritic arbor. Specifically, although individual physiological properties could be mediated by specific ion channels, these properties could be significantly regulated by other coexpressing channels/receptors, thereby contributing to functional robustness through degeneracy.

## Results

### Blocking KA channels enhanced neuronal intrinsic excitability and temporal summation across the somato-apical trunk

**Figure 1:**
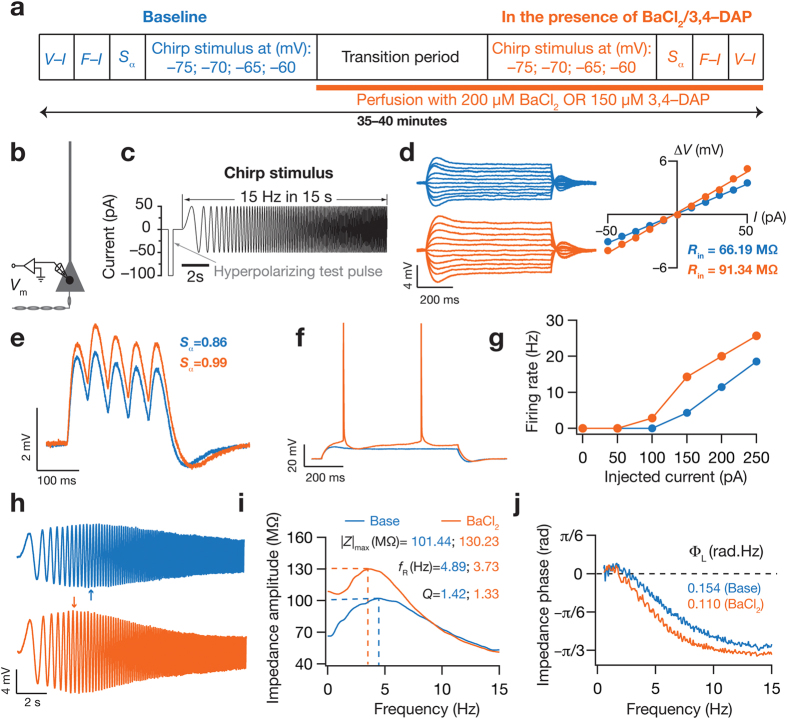
Typical experiment demonstrating the regulation of neuronal excitability and spectral tuning by KA channels. (**a**) Experimental protocol for assessing the effect of blocking KA channels on various physiologically relevant measurements. (**b**) Schematic of somato-apical trunk showing experimental setup and recording location. (**c**) *Chirp15* stimulus used for assessing intrinsic response dynamics and excitability. Neuron’s voltage response to initial hyperpolarizing test pulse of −100 pA amplitude was used for computing an estimate of input resistance, 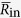, whereas the response to *Chirp15* stimulus was used to assess intrinsic spectral tuning. (**d**) *Left:* Voltage response of an example cell to constant current injection for 700 ms of varying amplitude, from −50 to +50 pA in steps of 10 pA, under baseline condition (blue) and after blocking KA channels using BaCl_2_ (orange). *Right: V*–*I* plot obtained from the traces shown in left. Input resistance, *R*_in_, was measured as slope of the linear fit to corresponding steady-state *V*-*I* curve. (**e**) Voltage traces in response to a train of five α-EPSCs at 20 Hz under baseline condition (blue) and after blocking KA channels (orange). 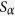: temporal summation strength. (**f**) Voltage traces in response to constant current injection of 100 pA for 700 ms under baseline conditions (blue) and after blocking KA channels (orange). (**g**) Firing rate profile of the example cell under baseline condition (blue) and after blocking KA channels (orange). (**h**) Example voltage traces in response to *Chirp15* stimulus under baseline condition (blue) and after blocking KA channels (orange). Arrow corresponds to the location of maximal response. (**i**) Impedance amplitude as a function of input current frequency derived from corresponding color matched traces shown in (**h**). *f*_R_: resonance frequency, *Q*: resonance strength, |*Z*|_max_: maximum impedance amplitude. (**j**) Impedance phase as a function of input current frequency derived from corresponding color matched traces shown in (**h**). Φ_L_: total inductive phase. All traces and measurements depicted in this figure were obtained from the same cell at the soma and recorded at −65 mV.

**Figure 2:**
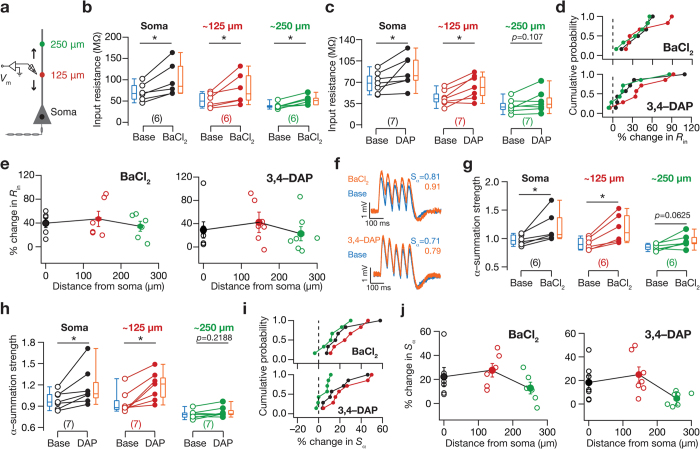
Blocking KA channels resulted in increased subthreshold intrinsic excitability and enhanced temporal summation across the somato-apical trunk. (**a**) Schematic of somato-apical trunk showing experimental setup for assessing the effect of blocking KA channels on various physiologically relevant measurements at various locations along the somato-apical trunk (up to ~300 μm). Local voltage responses at various locations along the somato-apical trunk were recorded in response to current stimuli injected through an electrode. Recording locations were binned into three sub-populations (soma, ~125 μm and ~250 μm) depending on their distance from the pyramidal cell layer, and the colored dots shown along the somato-apical trunk serve as codes for corresponding sub-populations in (**b–j**). (**b,c**) Population data (also depicted as quartiles) for the effect of blocking KA channels, using BaCl_2_ (**b**) or 3,4-DAP (**c**), on *R*_in_ for the three sub-populations of recording locations. *p < 0.05 Mann Whitney U test. (**d**) Cumulative probability of percentage change in *R*_in_ in response to blocking *A*-type K^+^ channels using BaCl_2_ (*top*) or 3,4-DAP (*bottom*). (**e**) Population data for percentage change in *R*_in_ after blocking KA channels, using either BaCl_2_ (*left*) or 3,4-DAP (*right*), plotted as a function of recording location. Open circles represent individual cells and filled circles represent the average values (mean ± SEM). (**f**) Voltage traces recorded from dendrites located at 240 μm (*top*) and 270 μm (*bottom*) away from soma in response to a train of five α-EPSCs at 20 Hz under baseline condition (blue) and after blocking KA channels (orange), using BaCl_2_ (*top*) and 3,4-DAP (*bottom*), respectively. (**g–j**) Same as (**b–e**) for temporal summation strength, *S*_*α*_. All measurements were obtained at −65 mV.

KA channels have been shown to regulate neuronal input resistance (*R*_in_) and action potential firing frequency of hippocampal CA1 pyramidal neuron somata^20^. However, it is not known if these somato-centric changes in excitability extend to dendritic locations, and if these changes in excitability extend to changes in temporal summation across the somatodendritic compartments. Given the high density of KA channels in CA1 pyramidal neuron dendrites^21^, and given that KA channels have been shown to regulate excitatory post synaptic potentials, EPSP^21^, we first explored the role of KA channels in regulating sub- and suprathreshold excitability across the somatoapical trunk of CA1 pyramidal neurons. To do this, we measured *R*_in_, action potential firing frequency and temporal summation strength (*S*_α_), before and after blocking KA channels (Fig. 1) using either 200 μM BaCl_2_^22^ or 150 μM 3,4-DAP^23^ in separate experiments, from soma or dendrites (up to ~300 μm from the soma; all recordings in this study were performed at physiological temperatures) of CA1 pyramidal neurons. We first assessed the subthreshold measures of excitability, and found that blocking KA channels significantly increased *R*_in_ across the somato-dendritic axis (Figs 1d and 2b,c; Tables S1 and S2), with percentage changes not significantly different along the somato-dendritic axis (Fig. 2d,e, BaCl_2_: *p* = 0.88 and 3,4-DAP: *p* = 0.38, Kruskal-Wallis rank sum test).

**Figure 3:**
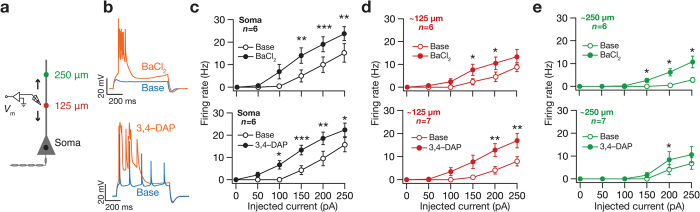
Blocking KA channels resulted in increased suprathreshold intrinsic excitability across the somato-apical trunk. (**a**) Same as Fig. 2a. (**b**) Voltage traces recorded from dendrites located at 240 μm (*top*; 200 pA current) and 270 μm (*bottom*; 250 pA current) away from the soma under baseline condition (blue) and after blocking KA channels (orange) using BaCl_2_ (*top*) and 3,4-DAP (*bottom*), respectively. Note that action potentials fired in bursts riding on a plateau after blocking KA channels. (**c–e**) Population plots of action potential firing frequency as a function of injected current amplitude at soma (**c**), dendrites around 125 μm (**d**) and dendrites around 250 μm (**e**) before (*open circles*) and after (*filled circles*) blocking KA channels using BaCl_2_ (*top*) or 3,4-DAP (*bottom*). *p < 0.05, **p < 0.005, ***p < 0.0005, paired Student’s *t* test. Data is presented as mean ± SEM. All measurements were obtained at −65 mV.

Next, analyzing the role of KA channels in regulating summation of α-EPSPs, we found that *S*_α_ increased after blocking KA channels with BaCl_2_ (Fig. 2g), with percentage changes not significantly different along the somato-dendritic axis (Fig. 2i,j, BaCl_2_: *p* = 0. 27, Kruskal-Wallis rank sum test; Table S1). On the other hand, although *S*_α_ increased when KA channels were blocked with 3,4-DAP (Fig. 2h), blockade-induced changes in *S*_α_ at distal dendrites were significantly lower than those at proximal dendrites and the somata (Fig. 2i,j, 3,4-DAP: *p* = 0.017, Kruskal-Wallis rank sum test, followed by Wilcoxon rank sum test: *p* = 0.32 for soma *vs*. 125 μm, *p* = 0.053 for soma *vs*. 250 μm and *p* = 0.007 for 125 μm *vs*. 250 μm; Table S2).

Turning to suprathreshold measures of excitability, we found that blocking KA channels significantly increased the firing frequency at various locations along the somato-dendritic axis (Fig. 3). With blockade of KA channels using either blocker, at higher current injections the pattern of action potential firing transitioned to burst firing in somatic recordings, whereas dendritic recordings showed an enhanced presence of plateau potentials^24^ (Fig. 3b). Specifically, after bath application of 3,4-DAP, 19 out of 21 recordings across the somato-apical axis showed robust burst firing with plateau potentials (soma: 7/7; ~125 μm: 7/7; ~250 μm: 5/7). On the other hand, bath application of BaCl_2_ was less efficient in inducing burst firing with plateau potentials, with only 8 out of 18 recordings made across the somato-apical axis displaying bursting with plateau potentials (soma: 0/6; ~125 μm: 4/6; ~250 μm: 4/6). Additionally, in a small percentage of recordings, the neuron switched to spontaneous firing after blocking KA channels (5/20 for BaCl_2_ and 9/28 for 3,4-DAP). Taken together, our results show that blocking KA channels results in significantly enhanced sub- and supra-threshold somatodendritic intrinsic excitability.

### Blocking KA channels significantly altered frequency-dependent response properties across the somato-apical trunk

**Figure 4:**
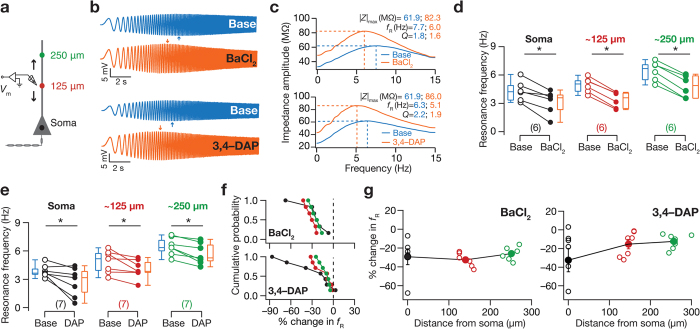
Blocking KA channels resulted in a decrease in resonance frequency across the somatoapical trunk. (**a**) Same as Fig. 2a. (**b**) Voltage traces recorded from dendrites located at 240 μm (*top*) and 270 μm (*bottom*) away from soma in response to the *Chirp15* stimulus under baseline condition (blue) and after blocking KA channels (orange), using BaCl_2_ (*top*) and 3,4-DAP (*bottom*), respectively. (**c**) Impedance amplitude plotted as functions of input current frequency derived from corresponding color-matched traces shown in (**b**), under baseline condition (blue) and after blocking KA channels (orange) using BaCl_2_ (*top*) or 3,4-DAP (*bottom*). (**d,e**) Population data (also depicted as quartiles) for the effect of blocking KA channels, using BaCl_2_ (**d**) or 3,4-DAP (**e**), on resonance frequency (*f*_R_) for the three sub-populations of recording locations. *p < 0.05 Mann Whitney U test. (**f**) Cumulative probability of percentage change in *f*_R_ in response to blocking *A*-type K^+^ channels using BaCl_2_ (*top*) or 3,4-DAP (*bottom*). (**g**) Population data for percentage change in *f*_R_ after blocking KA channels, using either BaCl_2_ (*left*) or 3,4-DAP (*right*), plotted as a function of recording location. Open circles represent individual cells and filled circles represent the average values (mean ± SEM).

Computational models have predicted that KA channels, despite being fast transient channels, could alter low-frequency spectral tuning in CA1 pyramidal neuron somata and their dendrites^2^,^7^. To test this prediction, we measured the responses of soma or dendrites to a chirp current (0–15 Hz in 15 s; Fig. 1c) stimulus before and after blocking KA channels (Figs 1 and 4b). Quantifying frequency-dependent response properties of neurons from the voltage responses to the chirp stimulus (Fig. 4c), we found that blocking KA channels resulted in a decrease in resonance frequency, *f*_R_, across the somato-apical trunk (Fig. 4d,e) (Tables S1 and S2), with percentage changes not significantly different across the somato-apical trunk (Fig. 4f,g, BaCl_2_: *p* = 0.28 and 3,4-DAP: *p* = 0.43 Kruskal-Wallis rank sum test).

**Figure 5:**
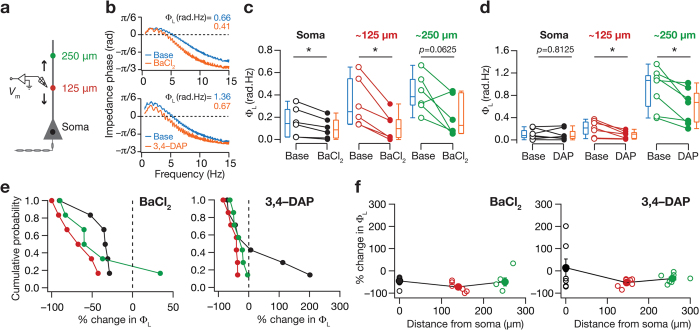
Blocking KA channels altered impedance phase and resulted in a decrease in total inductive phase across the somatoapical trunk. (**a**) Same as Fig. 2a. (**b**) Impedance phase plotted as functions of input current frequency derived from corresponding color-matched traces shown in Fig. 4a, under baseline condition (blue) and after blocking KA channels (orange) using BaCl_2_ (*top*) or 3,4-DAP (*bottom*). (**c,d**) Population data (also depicted as quartiles) for the effect of blocking KA channels, using BaCl_2_ (**c**) or 3,4-DAP (**d**), on Φ_L_ for the three sub-populations of recording locations. *p < 0.05 Mann Whitney U test. (**e**) Cumulative probability of percentage change in Φ_L_ in response to blocking *A*-type K^+^ channels using BaCl_2_ (*left*) or 3,4-DAP (*right*). (**f**) Population data for percentage change in Φ_L_ after blocking KA channels, using either BaCl_2_ (*left*) or 3,4-DAP (*right*), plotted as a function of recording location. Open circles represent individual cells and filled circles represent the average values (mean ± SEM). All measurements were obtained at −65 mV.

Turning to phase response dynamics (Fig. 5b), we found that blocking KA channels lead to a decrease in total inductive phase, Φ_L_^18^, at various locations along the somato-dendritic axis (Fig. 5c,d) (Tables S1 and S2) with percentage changes in this reduction very similar across all somato-apical locations (Fig. 5e,f, BaCl_2_: *p* = 0.15 and 3,4-DAP: *p* = 0.29 Kruskal-Wallis rank sum test). Together, our results demonstrate a critical role for KA channels in regulating theta-frequency spectral tuning and in frequency-dependent intrinsic phase response of hippocampal pyramidal neuron somata and dendrites. Importantly, the impact of two structurally distinct KA-channel blockers, BaCl_2_ and 3,4-DAP, resulted in very similar results (both qualitatively and quantitatively) in terms of the excitability (Figs 2 and 3) and frequency-dependent measurements (Figs 4 and 5), thereby strengthening our conclusions about the role of KA channels (Tables S1 and S2).

### Effect of blocking KA channels on intrinsic response dynamics was higher at depolarized potentials

**Figure 6:**
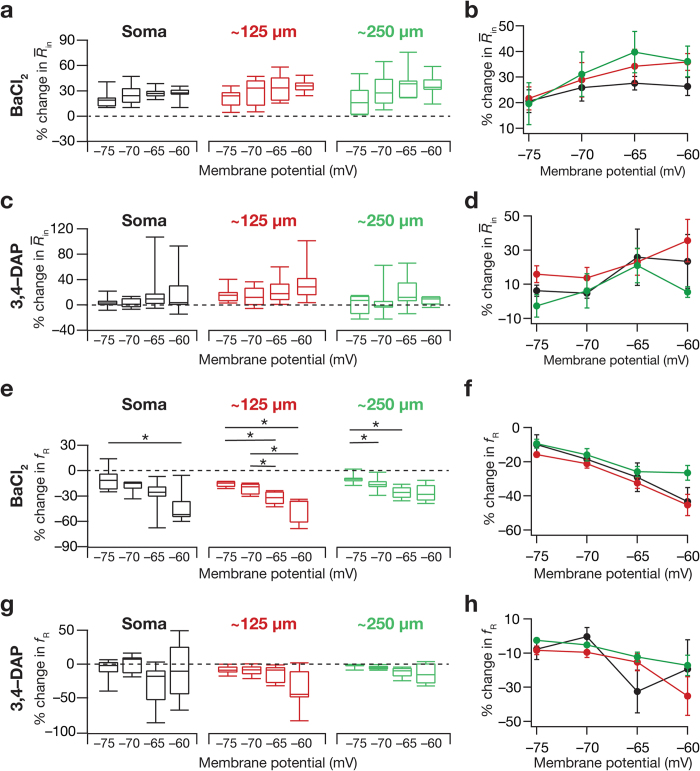
Blocking KA channels had a larger effect on resonance frequency at depolarized potentials. (**a**) Percentage change in input resistance (represented as quartiles) measured at different membrane potentials and for various recording locations after blocking KA channels using BaCl_2_ (Kruskal-Wallis rank sum test, *p* = 0.47 for input resistance at soma; *p* = 0.19 for input resistance at ~125 μm; *p* = 0.29 for input resistance at ~250 μm). (**b**) Percentage change (mean ± SEM) in input resistance after blocking KA channels using BaCl_2_ plotted as a function of membrane potential. (**c,d**) Same as (**a,b**) but employing 3,4-DAP to block KA channels (Kruskal-Wallis rank sum test, *p* = 0.54 for input resistance at soma; *p* = 0.40 for input resistance at ~125 μm; *p* = 0.55 for input resistance at ~250 μm). (**e**) Percentage change in *f*_R_ (represented as quartiles) measured at different membrane potentials and for various recording locations after blocking KA channels using BaCl_2_ (Kruskal-Wallis rank sum test, *p* < 0.05 for *f*_R_ at soma; *p* < 0.001 for *f*_R_ at ~125 μm; *p* < 0.01 for *f*_R_ at ~250 μm; followed by Mann-Whitney U test, *p < 0.05). (**f**) Percentage change (mean ± SEM) in *f*_R_ after blocking KA channels using BaCl_2_ plotted as a function of membrane potential. (**g,h**) Same as (**e,f**) but employing 3,4-DAP to block KA channels (Kruskal-Wallis rank sum test, *p* = 0.12 for *f*_R_ at soma; *p* = 0.30 for *f*_R_ at ~125 μm; *p* = 0.12 for *f*_R_ at ~250 μm).

As resonating properties depend heavily on membrane potential, and different channels alter excitability and resonance properties differentially at different membrane voltages depending on their activation-inactivation voltage ranges^7^,^12^,^14^,^18^,^25^, we employed voltage-dependence of physiological measurements as an additional tool to assess the specificity of the measurements to KA channel blockade. Specifically, hippocampal KA channels are active at depolarized voltages beyond −70 mV, with the window component of the KA current active in the voltage range between −70 mV to −20 mV^20^,^21^. Therefore, the impact of blocking KA channels on physiological measurements should be higher at more depolarized potentials than at hyperpolarized potentials (in the subthreshold range). Additionally, as steady-state measurements (like *R*_in_) are critically dependent on the window component of the KA current^7^,^20^, and an effect of KA channels on physiological measurements should be consistent with the channel activation range, we probed the impact of blocking KA channels on the voltage-dependence (range: −75 to −60 mV) of these measurements (Fig. 6, S1). We found that the impact of blocking KA channels on *f*_R_ was graded as a function of membrane voltage, with a significantly higher impact at depolarized voltages compared to their hyperpolarized counterparts (Fig. 6e–h, see Supplementary Fig. S1). Similar trends were observed in *R*_in_, |*Z*|_max_ and other measurements as well (Fig. 6a–d, see Supplementary Fig. S1), thereby providing an additional line of evidence that the observed changes were specific to blockade of KA channels. Specifically, as expected from the increased excitability that resulted from blocking KA channels (Fig. 2) and as predicted by computational models^2^, we found that the maximum impedance amplitude |*Z*|_max_ increased after KA-channel blockade (Fig. 4c, see Supplementary Figs S1f and S1l), with percentage differences not very different across locations (see Supplementary Fig. S1f).

### Barium chloride at lower concentrations was insufficient to alter intrinsic excitability or frequency-dependent response properties at depolarized potentials

**Figure 7:**
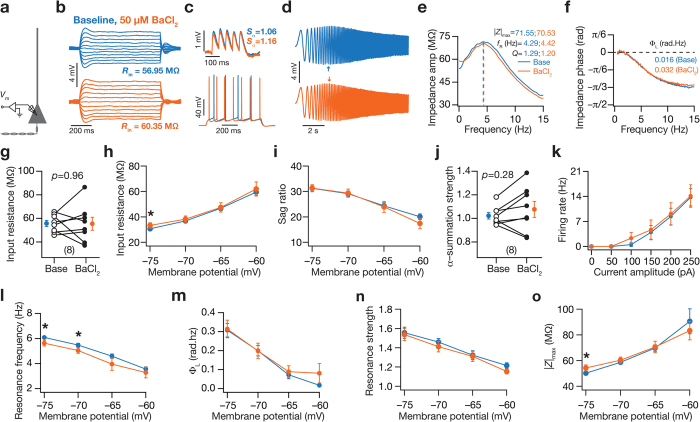
Bath application of 50 μM BaCl_2_ introduced small but significant differences in certain subthreshold measurements at hyperpolarized, but not depolarized, voltages. (**a**) Schematic showing experimental setup for assessing the effect of bath application of 50 μM BaCl_2_ on various physiologically relevant measurements at the soma. The experimental protocol was the same as that depicted in Fig. 1a, with the only difference being the reduction in the concentration of BaCl_2_ from 200 μM to 50 μM. (**b**) Voltage response of an example cell to constant current injection for 700 ms of varying amplitude, from −50 to +50 pA in steps of 10 pA, under baseline condition (blue) and after application of 50 μM BaCl_2_ (orange). (**c**) Top, voltage traces in response to a train of five α-EPSCs at 20 Hz under baseline condition (blue) and after application of 50 μM BaCl_2_ (orange). Voltage traces, in response to a pulse current of 200 pA for 700 ms, recorded from the soma under baseline condition (blue) and after application of 50 μM BaCl_2_ (orange). (**d**) Voltage traces recorded from the soma in response to the *Chirp15* stimulus under baseline condition (blue) and after application of 50 μM BaCl_2_ (orange). All traces in (**b–d**) were recorded from the same neuron. (**e,f**) Impedance amplitude (**e**) and phase (**f**) plotted as functions of input current frequency derived from corresponding color-matched traces shown in (**d**). (**g–o**) Population data (*n* = 8; mean ± SEM) for the effect of applying 50 μM BaCl_2_ on input resistance (**g**); input resistance (**h**) and sag ratio (**i**) as functions of voltage; temporal summation strength (**j**); action potential firing frequency for different current injections (**k**); and resonance frequency (**l**), total inductive phase (**m**), resonance strength (**n**) and maximal impedance amplitude (**o**) as functions of voltage. For (**g–o**), *p < 0.05 for paired Student’s *t* test, baseline measurements are depicted in blue and measurements obtained after application of 50 μM BaCl_2_ are plotted in orange.

BaCl_2_ is an established blocker of certain types of inward-rectifying potassium (KIR) channels^26^,^27^ at lower concentrations (50 μM). This implies that KIR channels were also blocked in our experiments with BaCl_2_, where we had employed BaCl_2_ at 200 μM to block KA channels^22^. What was the contribution of KIR channels to the changes (Figs 2, 3, 4, 5, 6, Fig. S1) in intrinsic excitability, spectral selectivity and phase tuning observed with BaCl_2_ as the pharmacological agent? Could the voltage-dependence of intrinsic measurements be explained with blockade of KIR channels? To answer these questions directly, we measured changes in intrinsic properties before and after application of 50 μM BaCl_2_, a concentration where BaCl_2_-induced blockade of KIR channels is more efficacious than that of KA channels. We found that 50 μM BaCl_2_ was insufficient to elicit significant differences in *R*_in_, firing frequency or temporal summation (Fig. 7b,c,g,j,k). Additionally, when we assessed intrinsic measurements as functions of membrane voltage, in striking contrast to our results with 200 μM BaCl_2_ (Fig. 6, Fig. S1), we found that 50 μM BaCl_2_ was insufficient to introduce significant changes in any of the measurements at depolarized potentials (Fig. 7h,i,k–o). Significant effects of applying 50 μM BaCl_2_ were confined to measurements at hyperpolarized voltages, specifically in 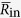 (Fig. 7h), *f*_R_ (Fig. 7l) and |*Z*|_max_ (Fig. 7o), thereby ruling out a role for KIR channels in introducing significant changes observed in depolarizing potentials (with 200 μM BaCl_2_). Together, these results argue against a significant role for KIR channels in eliciting changes (Figs 2, 3, 4, 5, 6; Fig. S1) observed with 200 μM BaCl_2_, thereby providing a critical line of evidence that the changes observed after the application of 200 μM BaCl_2_ were specific to the blockade of KA channels.

### Weak pairwise correlations between percentage changes in different measurements underscore the role of interactions among different ion channels

Computational models that have argued that functional homeostasis does not require or translate to individual channelostasis have shown that the impact of blocking KA channels on several measurements is variable, with changes in specific measurements depending critically on the expression profiles and properties of other channels^2^,^5^,^6^,^7^. Additionally, computational models have shown that different measurements are differentially dependent on different sets of ion channels^7^. Given such variability and spatiotemporal interactions with other channels, we postulated that pairwise correlations between percentage changes in different measurements obtained from the *same* cell be weakly correlated.

**Figure 8:**
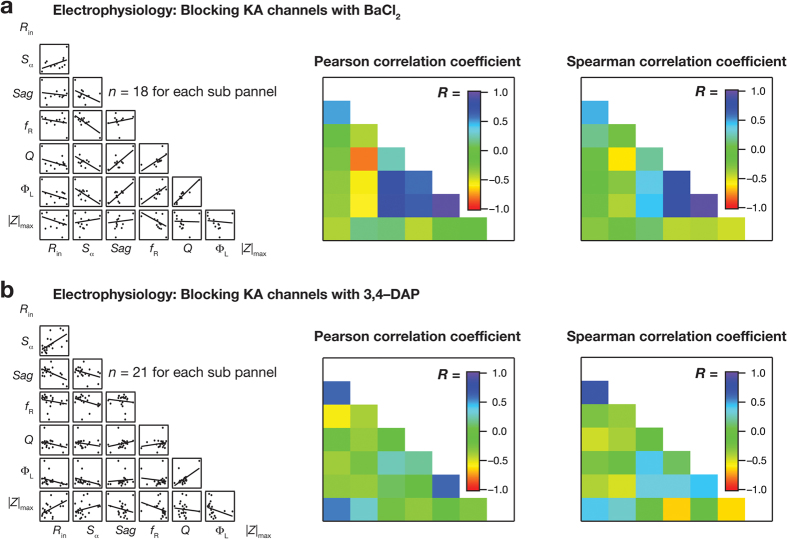
Weak pairwise correlations between intra-neuron percentage changes in different measurements after blockade of KA channels. (**a,b**) *Left*, Scatter plot matrix depicting the correlation between percentage changes among various measurements (at −65 mV) obtained from electrophysiological experiments using BaCl_2_ (**a**) or 3,4-DAP (**b**) to block KA channels. Color-coded matrix depicting the Pearson (*Center*) and Spearman (*Right*) correlation coefficient values for the corresponding scatter plots shown in the left. Percentage changes were pooled across all locations (a: *n* = 18; b: *n* = 21).

To test the postulate using electrophysiological data, we computed the correlation coefficients between percentage changes among various measurements in response to blocking KA channels, either using BaCl_2_ (Fig. 8a) or 3,4-DAP (Fig. 8b). We found that most pairwise correlation coefficients (both Pearson and Spearman) between percentage changes among various measurements were weak, with only a small percentage showing higher correlations (BaCl_2_; Fig. 8a and 3,4-DAP; Fig. 8b). As these correlations were computed from measurements obtained from the *same* soma/dendrite, these results cannot be attributed to the differential expression of KA channels. Instead, these results suggest that KA channels differentially contribute to different physiological measurements of a given neuron, and underscore the critical role of interactions among different ion channels in regulating individual physiological measurements.

## Discussion

The prime conclusion of this study is that the fast and transient KA channels play a critical role in regulating the theta-frequency spectral tuning map along the somatoapical trunk of hippocampal pyramidal neurons. The reduction in the tuning frequency, across the somato-apical trunk, effectuated by the blockade of KA channels also reflected as reductions in strength of spectral tuning and in total inductive phase. Additionally, extending previous somatocentric measurements of excitability, we also demonstrate that blocking KA channels significantly enhance input resistance, temporal summation strength, action potential firing frequency, propensity of plateau potentials and burst firing, and frequency-dependent measurements of neuronal gain across the somatodendritic arbor. Finally, assessing correlations between percentage changes in several measurements from the *same* neuron, we demonstrate that different measurements are differentially regulated by KA channels, emphasizing the importance of cross-channel interactions predicted by modeling studies.

### Implications for the regulation of somatodendritic excitability and spectral tuning by KA channels

From the neuronal plasticity perspective, our results demonstrating the role of KA channels in regulating intrinsic excitability across the somatodendritic axis would directly imply that the neuron would be subjected to significant metaplasticity as a consequence of absence of or plasticity in KA channels at any or all of these locations^9^,^28^,^29^,^30^,^31^. Furthermore, spiking occurring at different phases of membrane potential oscillations have profound effect on inducing long-term potentiation *vs*. depression^32^. A direct consequence of the ability of KA channels to alter the phase of afferent inputs is that the somatic spike phase would be different, implying potential differences in such spike-phase dependent synaptic plasticity. Finally, such alterations to synaptic plasticity profiles are consequent to changes in excitability that translate to changes in calcium influx into the cellular compartment where plasticity is induced. As intrinsic plasticity profiles are also dependent on calcium influx^33^,^34^,^35^, this altered calcium influx could additionally induce metaplasticity in intrinsic plasticity profiles across the dendritic axis.

How would the ability of KA channels to introduce changes in somatodendritic spectral tuning and intrinsic phase response alter neuronal information processing? First, given the significant relationships that have been established between neuronal spike initiation dynamics and subthreshold resonance^36^,^37^, and given that KA channels can alter subthreshold somatodendritic spectral tuning (Fig. 4), it stands to reason that KA channels would significantly alter location-dependent spike initiation dynamics of a neuron. Additionally, subthreshold dendritic resonance and dendritic spike generation have been critically implicated in high-frequency coincidence detection capabilities. Therefore, it would follow that the ability of KA channels to change spectral tuning and local excitability would alter the integration *vs.* coincidence detection (class 1 *vs*. class 2/3 excitability) capabilities of a neuron^8^,^36^,^37^. The voltage-dependence of KA channels in regulating these properties (Fig. 6), in conjunction with voltage-dependent regulation by other channels^7^,^12^,^18^, provides neurons with additional degrees of freedom in robustly regulating spectral tuning and excitability in neuronal compartments. Further, computational models have shown that the window current of KA channels forms the biophysical basis for KA-dependent regulation of low-frequency impedance properties^7^. Future studies could further assess the role of this window component^20^,^21^ in regulating spike initiation dynamics and neuron coincidence detection capabilities^37^.

Second, it has been shown that neurons possess a theta-range crossover frequency where the phase of the incoming oscillatory inputs is normalized at the soma, irrespective of the location of input origin along the somatoapical trunk. This oscillatory synchrony was demonstrated to be a consequence of the differential phase lead introduced by the somatoapical gradient in HCN channels^38^. Our results that KA channels could substantially modulate impedance phase profile (Fig. 5) clearly suggest that KA channel gradients, apart from and in conjunction with HCN channels, could contribute to the emergence and regulation of oscillatory synchrony. Future studies should explore if the maintenance of oscillatory synchrony requires HCN channels to be maintained at specific expression levels, or if interactions among several channels would imply degeneracy with reference to oscillatory synchrony.

Finally, several lines of recent evidence suggest a critical role for sub- and suprathreshold dendritic ion channels in regulating the amplitude and phase of local field potentials (LFP) in different frequency bands^39^,^40^,^41^. Given our results that KA channels could alter neuronal excitability and impedance phase (Figs 2, 3, 4, 5), it is clear that changes in KA-channel density and properties could significantly alter LFP amplitude and phase. These changes could be consequent to the altered prevalence of plateau potentials or to changes in impedance phase of a wide range of input frequencies observed with changes in KA channels (Figs 3 and 5). Additionally, intracellular spiking has been shown to contribute to LFPs in epsilon band^40^. As KA channels regulate action potential properties, including the half width of backpropagating action potentials^21^,^42^, the presence/plasticity of KA channels could regulate LFPs in the epsilon band. Such changes in LFP amplitude/phase could be localized or widespread along the hippocampal neuropil^41^, depending on the localization profile of KA-channel plasticity, which has been demonstrated to be localized in certain cases^34^ but is more widespread with channelopathies^1^.

### Implications for differential regulation of neuronal properties by multiple channel conductances

Theta-frequency spectral tuning in hippocampal pyramidal neurons has primarily been attributed to slow channels such as *M*-type K^+^ and HCN channels^12^,^13^,^14^. Although these slow channels mediate low frequency spectral tuning and theta-frequency phase lead, owing to their unique spectral response characteristics^17^,^18^, our results clearly establish that KA channels provide an additional mechanism for regulating low frequency spectral tuning and intrinsic phase lead across the somatodendritic arbor. Apart from changes in spectral tuning and phase response, our results also demonstrate that KA channels alter input resistance, firing frequency, sag ratio, temporal summation and maximal impedance amplitude, properties that are known to be regulated by other channels as well^12^,^18^,^25^,^30^,^33^,^43^,^44^,^45^. What are the implications for multiple channels regulating the same physiological measurements? Several recent studies spanning multiple systems (including hippocampal pyramidal neurons) have shown that the relationship between channel distributions and functional measurements manifests degeneracy^2^,^5^,^6^,^7^,^8^,^9^,^10^,^46^. A critical requirement for neurons to exhibit such degeneracy is that several mechanisms contribute to the regulation of specific physiological properties. Our results demonstrate that KA channels critically modulate each of the several physiological measurements, revealing previously unknown functions of these channels, and constitute a mechanism to fulfill this requirement for establishing robustness through degeneracy^2^,^7^.

Additionally, through a novel analysis that assesses correlations between variability in percentage changes (as a consequence of KA channel blockade) in different measurements from the same cell, we demonstrate that KA channels differentially regulate different physiological measurements (Fig. 8). This is consistent with conclusions from modeling studies that have shown that different measurements have differential dependencies on any given channel, in a manner that is also reliant on spatial and kinetic interactions among different channels, their expression profiles and properties^2^,^5^,^6^,^7^,^8^,^9^. These results suggest that such uncorrelated changes in different measurements provide more degrees of freedom for how each channel property alters any physiological behavior, further contributing to robust homeostasis through degeneracy. Finally, the existence of such degeneracy implies that the normalization of unitary synaptic potentials^47^, temporal summation^48^ and somatic theta-phase^38^ (see above) in hippocampal pyramidal neurons is regulated by the collective set of channels and receptors expressed in the dendrites, and blockade of any of the several dendritic ion channels would differentially alter such normalization.

### Computational modeling *vs*. electrophysiological experiments: Consistencies and contrasts

The direction and percentage changes observed in *f*_R_, |*Z*|_max_ and *R*_in_ after blocking KA channels (Figs 2, 3, 4, see Supplementary Fig. S1) were consistent with results from computational modeling where the KA conductance was eliminated^2^,^7^,^49^. However, the direction of changes in *Q* and Φ_L_ derived from electrophysiological recordings (Fig. 5, see Supplementary Fig. S1) were in striking contrast with those obtained from computational modeling^2^,^7^. Specifically, whereas modeling studies showed that removing the KA conductance results in increases in *Q* and Φ_L_^2^,^7^, our results with pharmacological blockade of KA channels led to decreases in both measurements. These consistencies and contrasts were common for pharmacological blockade with either BaCl_2_ or 3,4-DAP, therefore potentially ruling out a role for pharmacological non-specificities. The exact reasons behind the observed discrepancies with reference to *Q* and Φ_L_ are unknown. However, the modeling study did not incorporate all ion channels, and several lines of evidence suggest that ion channels interact with each other and blockade of one type of ion channels could lead to significant changes in properties of other ion channels through structural or activity-independent interactions^50^,^51^,^52^,^53^,^54^, possibilities that should be explored in future studies.

Although 3,4-DAP and BaCl_2_ have been shown to be blockers of KA channels^22^,^55^,^56^, there are established non-specificities associated with these blockers, at concentrations employed in our study. Specifically, whereas 3,4-DAP is known to block delayed rectifier potassium (KDR) channels^23^, BaCl_2_ is an established blocker of certain types of KIR channels^26^,^27^. We note that the prime conclusion of our study with reference to the regulation of theta-frequency resonance and phase lead by KA channels is consistent across both blockers. Apart from this, there are additional lines of evidence that emphasize our conclusions that the observed changes are specific to blockade of KA channels. First, KDR channels in hippocampal pyramidal neurons display a more depolarized activation compared to the range where our impedance measurements were performed. Specifically, whereas our measurements were performed in the subthreshold voltage range (−80 to −55 mV), the half-maximal activation voltage of KDR channels in these neurons is around +15 mV, with the open-probability of these channels remaining at zero until around −30 mV^21^. Second, modeling studies that employed channel kinetics from these neurons have clearly shown that resonance frequency and other impedance-related measurements are not dependent on KDR channels at subthreshold voltage ranges^2^. Together, these imply that the KDR channels are not active in the voltage range employed for all subthreshold measurements, and together with their fast kinetics would not contribute to theta-frequency spectral tuning at these voltage ranges. Although this analysis on the independence of our observations on KDR channels would hold for subthreshold measurements, the non-specific blockade of KDR channels by 3,4-DAP could significantly impact our supra-threshold measurements. Specifically, it is possible that such non-specific blockade (of KDR channels by 3,4-DAP) contributes to the enhanced burst propensity observed with 3,4-DAP compared to treatment with BaCl_2_. Finally, our results also argue against the involvement of KIR channels in determining sub-threshold spectral tuning properties. Specifically, we have shown that application of 50 μM BaCl_2_, a concentration at which KIR channels are more efficaciously blocked, was insufficient to introduce significant changes in intrinsic measurements at depolarized potentials (Fig. 7), which is in striking contrast to our results with 200 μM BaCl_2_ where KA channels are also blocked (Fig. 6; Fig. S1). Together, despite known non-specificities associated with these blockers, our experimental design involving independent sets of experiments with each blocker and the voltage-dependent properties of channels that are affected by these blockers present clear lines of evidence for the observed effects to be specific to blockade of KA channels.

In summary, consistent with our computational predictions^2^,^7^, we found that blocking KA channels resulted in a significant reduction in resonance frequency and significant increases in input resistance, impedance amplitude and action-potential firing frequency across the somato-apical trunk. Importantly, within the subthreshold range of voltages, KA channels were more effective in altering spectral tuning at more depolarized potentials compared to hyperpolarized voltages, providing neuronal compartments with voltage-dependent control of spectral tuning. Furthermore, across all measured locations, blocking KA channels enhanced temporal summation of postsynaptic potentials and significantly reduced total inductive phase that characterizes theta band lead in impedance phase. Finally, pair-wise correlations between intraneuronal percentage changes (consequent to blockade of KA channels) in different measurements were mostly weak, suggesting differential regulation of different physiological properties by KA channels. Our results unveil a pivotal role for fast transient channels in regulating theta-frequency spectral tuning and intrinsic phase response, and suggest that degeneracy with reference to several coexisting functional maps is mediated by cross-channel interactions across the active dendritic arbor. Future studies could also focus on degeneracy in the complementary form of theta-frequency spectral tuning that is mediated by the *M*-type potassium current at more depolarized potentials^12^,^13^,^14^,^57^. Finally, although our focus has been on spectral tuning in hippocampal neurons, these results on degeneracy are extendable to cortical neurons where spectral tuning is known to be mediated by similar set of ionic conductances^16^,^58^.

## Methods

All electrophysiological procedures were similar to those reported in previous studies^25^,^59^, and are detailed below.

### Ethics statement

All experiments reported in this study were performed in strict adherence to the protocols that were approved by the Institute Animal Ethics Committee (IAEC) of the Indian Institute of Science, Bangalore and The University of Texas at Austin Institutional Animal Care and Use Committee (IACUC).

### Slice preparation

5–9 weeks old male Sprague-Dawley rats were used in this study. Rats were anesthetized by intraperitoneal injection of a mixture of ketamine and xylazine. Under deep anesthesia, determined by cessation of toe-pinch reflex, rats were transcardially perfused with ice-cold cutting solution containing (in mM) 210 sucrose, 2.5 KCl, 1.25 NaH_2_PO_4_, 25 NaHCO_3_, 0.5 CaCl_2_, 7 MgCl_2_, 7 dextrose and 3 Na-pyruvate (all from Sigma Aldrich). After transcardial perfusion, rats were decapitated quickly and the brain was surgically removed in the presence of ice-cold cutting solution. 350-μm thick near-horizontal middle (Bregma −6.5 mm to −5.1 mm) hippocampal slices were prepared using VT1000P vibratome (Leica) in the presence of oxygenated ice-cold cutting solution. Slices were submerged in a holding chamber containing oxygenated chamber solution composed of (in mM): 125 NaCl, 2.5 KCl, 1.25 NaH_2_PO_4_, 25 NaHCO_3_, 1 CaCl_2_, 2 MgCl_2_, 10 dextrose and 3 Na-pyruvate, and were incubated at 34 °C for 10–20 minutes and then at room temperature for at least 45 minutes before recording.

### Electrophysiology

Slices were transferred to the recording chamber and were continuously perfused with oxygenated artificial cerebrospinal fluid (ACSF) containing (in mM) 125 NaCl, 3 KCl, 1.25 NaH_2_PO_4_, 25 NaHCO_3_, 2 CaCl_2_, 1 MgCl_2_, 10 dextrose. Slices were visualized using 63× water immersion lens through a Dodt contrast microscope (Carl Zeiss Axioexaminer). Visually identified somatic or dendritic whole cell current clamp recordings were made from CA1 pyramidal neurons using a BVC-700A (Dagan Inc.) amplifier. Data acquisition was done using custom-written software in the Igor Pro environment (Wavemetrics) and signals were digitized at 10 kHz using an ITC18 interface (HEKA). For recordings involving action potentials, signals were sampled at 40 kHz. In performing somatic or dendritic whole cell current clamp recordings, borosilicate glass capillaries (1.5-mm outer diameter and 0.86-mm inner diameter; Sutter Instruments) were pulled using a P-97 Flaming/Brown micropipette puller (Sutter Instruments). Glass pipettes were filled with intracellular recording solution containing (in mM) 120 K-gluconate, 20 KCl, 10 HEPES, 4 NaCl, 4 Mg-ATP, 0.3 Na_2_-GTP and 7 K_2_-phosphocreatine, pH 7.3 adjusted with KOH. In the presence of this intracellular recording solution, glass pipettes with 3–6 MΩ and 6–9 MΩ resistance were used for somatic and dendritic recordings, respectively. Series resistance was monitored and compensated online using the bridge-balance circuit of the amplifier. Experiments were discarded only if the initial resting membrane potential was more depolarized than −59 mV, if series resistance rose above 30 MΩ (for somatic recordings) or 45 MΩ (for dendritic recordings), or if there were fluctuations in temperature during the course of the experiment. All recordings were performed at physiological temperatures, around 32–34 °C (employing an inline heater from Warner Instruments). Voltages have not been corrected for the liquid junction potential, which was experimentally determined to be ~8 mV. All recordings were performed in the presence of a cocktail of synaptic blockers added to ACSF, containing (in μM) either 10 CNQX, 50 d,l-APV, 2 CGP55845,10 (+)bicuculin and 10 picrotoxin (all blockers from Allied Scientific) or 20 DNQX, 25 d-APV (both from Alomone Labs), 5 CGP55845 and 2 Gabazine (both from Allied Scientific). *A*-type K^+^ channels were blocked either by adding 200 μM of BaCl_2_^22^ or 150 μM of 3,4-DAP^23^,^55^ (both from Sigma-Aldrich) to the ACSF containing synaptic blockers.

### Electrophysiological data analysis

Physiologically relevant measurements^12^,^18^,^25^,^59^ were derived from the electrophysiological data recorded employing procedures described above. Specifically, input resistance (*R*_in_) was computed from a plot of steady-state voltage deflections in response to various levels of current injection (−50 to +50 pA in steps of 10 pA) *vs.* the injected current amplitude (the *V*-*I* curve). The slope of a linear fit to the *V*-*I* curve formed the *R*_in_. The single-pulse estimate of the input resistance (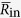) was computed as the ratio of the steady-state voltage deflection induced by a 100-pA hyperpolarizing pulse current.

Percentage sag was computed from the voltage response to a hyperpolarizing 100 pA current pulse and was defined as 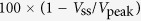, where *V*_ss_ was the steady state voltage deflection from baseline and *V*_peak_ was the peak voltage deflection from baseline. To assess temporal summation strength, *S*_*α*_, α-excitatory postsynaptic potentials (α-EPSP) were evoked by current injection (α-EPSC) of the form *I*_*α*_ = *I*_max_*t*.exp(−*αt*) with α = 0.1 ms^−1^. A train of five α-EPSCs was injected at 20 Hz and the ratio of the fifth α-EPSP amplitude to that of the first α-EPSP amplitude formed *S*_*α*_. Firing rate profile (*F*-*I* curve) was generated by injecting a current pulse of various amplitudes (0–250 pA, steps of 50 pA) for 700 ms. Action potential firing rate was computed by extrapolating the number of spikes obtained during the 700 ms current injection period to 1 s, and was plotted against the injected current amplitude.

To assess intrinsic frequency-dependent response properties of the neuronal compartment being recorded, a linear chirp current stimulus (*Chirp15*), spanning 0–15 Hz in 15 s, was injected and the corresponding voltage response was recorded. The Fourier transform of the voltage response of the neuronal compartment to the chirp stimulus was divided by the Fourier transform of injected current (*Chirp15*) to obtain complex-valued impedance *Z*(*f* ), as a function of frequency *f*. The impedance amplitude profile (ZAP) was computed as the magnitude of this impedance, defined as:


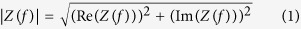


where Re(*Z*(*f* )) and Im(*Z*(*f* )) are the real and imaginary parts of the impedance *Z*(*f* ). The maximum value of |*Z*(*f* )| was denoted as |*Z*|_max_ and the frequency at which the impedance amplitude reached its maximum was defined as the resonance frequency (*f*_R_) of the neuronal compartment. Resonance strength (*Q*) was defined as the ratio of the maximum impedance amplitude (|*Z*|_max_) to the impedance amplitude at 0.5 Hz (|*Z*(0.5)|). The impedance phase profile (ZPP) was computed as:


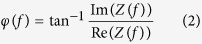


Total inductive phase was defined as the area under the inductive part of the ZPP:


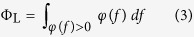


All data analyses were performed using custom-written software in Igor Pro (Wavemetrics), and statistical analyses were performed using the R computing package^60^. Details of statistical analyses performed for this study are provided in supplementary Tables S1 and S2.

### Computing correlation matrices of percentage changes in different measurements after blocking KA channels

Percentage changes in each of the 7 measurements were plotted against other measurements from the *same* neuron (intraneuronal), with recordings from different somato-dendritic locations pooled together. Pearson and Spearman correlation coefficients for each pair was computed using statistical computing language R^60^.

## Additional Information

**How to cite this article**: Rathour, R. K. *et al*. Transient potassium channels augment degeneracy in hippocampal active dendritic spectral tuning. *Sci. Rep.* **6**, 24678; doi: 10.1038/srep24678 (2016).

## Supplementary Material

Supplementary Information
