# Supplementary material for: Transient potassium channels augment degeneracy in hippocampal active dendritic spectral tuning
Source: Sci Rep. 2016 Apr 20;6:24678. doi: 10.1038/srep24678 (PMC4837398; doi:10.1038/srep24678)
Supplement: Supplementary Information [file srep24678-s1.pdf]

Transient potassium channels augment degeneracy in hippocampal active dendritic spectral tuning

Rahul Kumar Rathour[1], Ruchi Malik[2] and Rishikesh Narayanan[1]

[1]Cellular Neurophysiology Laboratory, Molecular Biophysics Unit, Indian Institute of Science, Bangalore, India.
[2]Center for Learning and Memory, The University of Texas at Austin, Austin, TX, USA.

SUPPLEMENTARY INFORMATION

Supplementary Figure S1 2
Supplementary Table S1 3
Supplementary Table S2 4

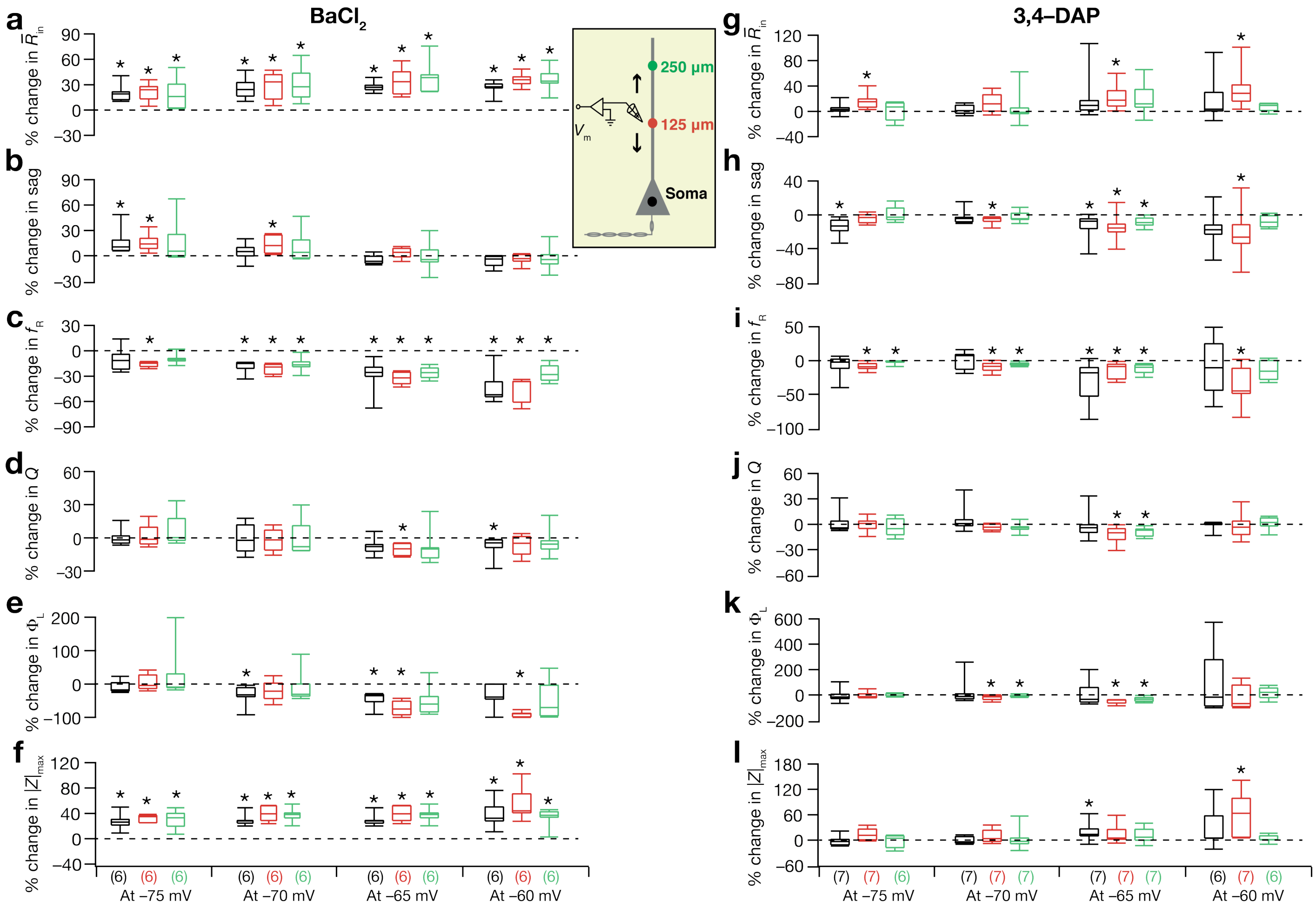

Supplementary Figure S1. Voltage-dependence of changes observed in various measurements after blocking KA channels. Data presented in (a–f) and (g–l) were derived from experiments using 200 µM of $BaCl_2$ and 150 µM of 3,4–DAP, respectively, for blocking KA channels. For all panels, percentage change (represented as quartiles) in the respective measurements after blocking KA channels is depicted for three locations and for four different membrane potentials. Asterisk on a given box plot denotes that the measurement values under baseline condition were significantly ($p < 0.05$, Mann-Whitney U test) different from those measured after blocking KA channels. The central inset provides the color code for the three subpopulations of neurons in (a–l).

Supplementary Table S1: Experimental measurements obtained from hippocampal CA1 pyramidal neurons before and after 200 µM $BaCl_2$ application. Data is presented as mean ± SEM. All measurements were obtained at –65 mV. *t*-test: Student's *t* test; MW U test: Mann-Whitney U test.

**Measurements obtained at soma (*n*=6)**				
**Measurements**	**Before $BaCl_2$**	**After $BaCl_2$**	***t*-test**	**MW U test**
Input resistance (MΩ)	70.87 ± 8.46	100.48 ± 15.95	$p<0.05$	$p<0.05$
Firing frequency (Hz)	Increased after $BaCl_2$		Figure 3	—
Temporal summation strength	0.97 ± 0.04	1.2 ± 0.11	$p<0.05$	$p<0.05$
Sag ratio	29.11 ± 1.82	27.62 ± 1.48	$p>0.05$	$p>0.05$
Resonance frequency (Hz)	4.31 ± 0.44	3.14 ± 0.51	$p<0.05$	$p<0.05$
Resonance strength	1.40 ± 0.05	1.30 ± 0.07	$p>0.05$	$p>0.05$
Total inductive phase (Hz.rad)	0.16 ± 0.05	0.10 ± 0.04	$p<0.05$	$p<0.05$
Maximum impedance amplitude (MΩ)	97.64 ± 9.71	136.23 ± 15.89	$p<0.05$	$p<0.05$
**Measurements obtained at ~125 µm (*n*=6)**				
Input resistance (MΩ)	51.84 ± 6.71	78.26 ± 14.76	$p<0.05$	$p<0.05$
Firing frequency (Hz)	Increased after $BaCl_2$		Figure 3	—
Temporal summation strength	0.90 ± 0.04	1.17 ± 0.10	$p<0.05$	$p<0.05$
Sag ratio	33.67 ± 1.14	34.80 ± 1.63	$p>0.05$	$p>0.05$
Resonance frequency (Hz)	4.94 ± 0.32	3.37 ± 0.34	$p<0.05$	$p<0.05$
Resonance strength	1.50 ± 0.066	1.36 ± 0.09	$p<0.05$	$p<0.05$
Total inductive phase (Hz.rad)	0.33 ± 0.09	0.12 ± 0.05	$p<0.05$	$p<0.05$
Maximum impedance amplitude (MΩ)	73.26 ± 8.25	102.73 ± 12.87	$p<0.05$	$p<0.05$
**Measurements obtained at ~250 µm (*n*=6)**				
Input resistance (MΩ)	38.86 ± 3.03	51.96 ± 4.57	$p<0.05$	$p<0.05$
Firing frequency (Hz)	Increased after $BaCl_2$		Figure 3	—
Temporal summation strength	0.85 ± 0.02	0.96 ± 0.05	$p<0.05$	$p>0.05$
Sag ratio	34.88 ± 1.40	34.33 ± 2.15	$p>0.05$	$p>0.05$
Resonance frequency (Hz)	6.43 ± 0.42	4.81 ± 0.47	$p<0.05$	$p<0.05$
Resonance strength	1.57 ± 0.06	1.45 ± 0.11	$p>0.05$	$p>0.05$
Total inductive phase (Hz.rad)	0.41 ± 0.07	0.20 ± 0.07	$p<0.05$	$p>0.05$
Maximum impedance amplitude (MΩ)	58.75 ± 4.27	82.03 ± 5.75	$p<0.05$	$p<0.05$

Supplementary Table S2: Experimental measurements obtained from hippocampal CA1 pyramidal neurons before and after 150 µM 3,4–DAP application. Data is presented as mean ± SEM. All measurements were obtained at –65 mV. *t*-test: Student's *t* test; MW U test: Mann-Whitney U test.

**Measurements obtained at soma (*n*=7)**				
**Measurements**	**Before DAP**	**After DAP**	***t*-test**	**MW U test**
Input resistance (MΩ)	69.38 ± 6.49	88.30 ± 9.70	p<0.05	p<0.05
Firing frequency (Hz)	Increased after 3,4 DAP		Figure 3	—
Temporal summation strength	0.99 ± 0.05	1.17 ± 0.10	p<0.05	p<0.05
Sag ratio	24.59 ± 2.36	20.94 ± 2.24	p>0.05	p<0.05
Resonance frequency (Hz)	3.86 ± 0.24	2.71 ± 0.58	p<0.05	p<0.05
Resonance strength	1.27 ± 0.05	1.24 ± 0.07	p>0.05	p>0.05
Total inductive phase (Hz.rad)	0.11 ± 0.04	0.10 ± 0.04	p>0.05	p>0.05
Maximum impedance amplitude (MΩ)	96.01 ± 8.67	113.35 ± 9.81	p<0.05	p<0.05
**Measurements obtained at ~125 µm (*n*=7)**				
Input resistance (MΩ)	44.37 ± 4.74	62.37 ± 7.40	p<0.05	p<0.05
Firing frequency (Hz)	Increased after 3,4 DAP		Figure 3	—
Temporal summation strength	0.95 ± 0.06	1.18 ± 0.08	p<0.05	p<0.05
Sag ratio	32.45 ± 2.06	27.25 ± 1.81	p<0.05	p<0.05
Resonance frequency (Hz)	4.86 ± 0.45	4.08 ± 0.38	p<0.05	p<0.05
Resonance strength	1.49 ± 0.08	1.29 ± 0.05	p<0.05	p<0.05
Total inductive phase (Hz.rad)	0.21 ± 0.05	0.09 ± 0.03	p<0.05	p<0.05
Maximum impedance amplitude (MΩ)	62.72 ± 5.01	71.98 ± 6.39	p>0.05	p>0.05
**Measurements obtained at ~250 µm (*n*=7)**				
Input resistance (MΩ)	31.10 ± 4.18	38.46 ± 4.57	p>0.05	p>0.05
Firing frequency (Hz)	Increased after 3,4 DAP		Figure 3	—
Temporal summation strength	0.78 ± 0.02	0.82 ± 0.02	p>0.05	p>0.05
Sag ratio	43.25 ± 3.03	39.38 ± 2.23	p<0.05	p<0.05
Resonance frequency (Hz)	6.49 ± 0.36	5.71 ± 0.42	p<0.05	p<0.05
Resonance strength	1.86 ± 0.11	1.68 ± 0.08	p<0.05	p<0.05
Total inductive phase (Hz.rad)	0.90 ± 0.14	0.60 ± 0.12	p<0.05	p<0.05
Maximum impedance amplitude (MΩ)	57.20 ± 7.66	62.62 ± 6.77	p>0.05	p>0.05
